# Nonanal modulates oviposition preference in female *Helicoverpa assulta* (Lepidoptera: Noctuidae) via the activation of peripheral neurons

**DOI:** 10.1002/ps.5870

**Published:** 2020-05-10

**Authors:** Chan Wang, Guannan Li, Changjian Miao, Man Zhao, Bing Wang, Xianru Guo

**Affiliations:** ^1^ College of Plant Protection Henan Agricultural University Zhengzhou China; ^2^ State Key Laboratory for Biology of Plant Diseases and Insect Pests Institute of Plant Protection, Chinese Academy of Agricultural Sciences Beijing China

**Keywords:** *Helicoverpa assulta*, *Nicotiana tabacum*, nonanal, oviposition preference, odorant receptor neuron, basiconic sensilla

## Abstract

**BACKGROUND:**

The insect olfactory system can recognize odorants for feeding, courtship, oviposition and avoiding natural enemies. Odorant cues from host plants play important roles in insect behaviours. Tobacco (*Nicotiana tabacum*) is the main cultivated host of the oriental tobacco budworm *Helicoverpa assult*. Volatiles of tobacco plants attract and stimulate oviposition in female moths. However, it is still not known how female *H. assulta* recognize tobacco volatiles and which odorant compounds are used as oviposition cues.

**RESULTS:**

We detected 14 volatile compounds emitted from a tobacco plant during vegetative growth, using gas chromatography–mass spectrometry. Electroantennogram tests indicated that eight of the 14 compounds induced responses in female *H. assulta.* Among these eight volatiles, nonanal greatly increased oviposition preference. Single‐sensillum recording (SSR) results showed that many neurons housed in three types of short basiconic sensilla and four types of long basiconic sensilla responded to nonanal and heptanal as its structural analogue. The responses to nonanal were significantly stronger than those to the other compounds. Nonanal was the main ligand of OR67, an odorant receptor from *H. assulta*. This was demonstrated using an *in vitro Xenopus* oocytes expression system that supported the SSR results.

**CONCLUSION:**

Nonanal is a key signal volatile of tobacco plants that attracts female *H. assulta* moths to oviposit. © 2020 The Authors. *Pest Management Science* published by John Wiley & Sons Ltd on behalf of Society of Chemical Industry.

## Graphical abstract

The main cultivated host of the oligophage oriental tobacco budworm *Helicoverpa assulta* is tobacco (*Nicotiana tabacum*). Volatiles of tobacco plants attract and stimulate oviposition in female moths.

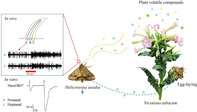

## 1 INTRODUCTION

The insect olfactory system plays an important role in many aspects of insect biology and ecology, including natural enemy avoidance, host preference, mate location and oviposition.^1^, ^2^ Insects can perceive odorants such as sex pheromones,^3^ aggregation pheromones,^4^ alarm pheromones^5^ and host‐plant volatiles^6^, ^7^, ^8^ through their olfactory system. Many proteins are involved in the olfactory system, including odorant‐binding proteins (OBPs),^9^ odorant receptors (ORs),^10^ sensory neuron membrane proteins (SNMPs)^11^ and odorant‐degrading enzymes (ODEs);^12^, ^13^ of these, ORs form the core of odorant detection.^14^

ORs are classic seven‐transmembrane proteins located on the dendritic membrane of odorant receptor neurons (ORNs) within specialized sensory sensilla.^15^ ORs are responsible for the sensitivity and specificity of olfactory sensory neurons by mediating the binding of specific odour molecules with olfactory neurons, and are thus important in the exchange of information between insects and their external environment.^16^, ^17^, ^18^, ^19^ ORs usually work together with a conserved odorant receptor co‐receptor (Orco).^19^, ^20^, ^21^, ^22^ The OR/Orco complex works by converting semiochemical signals into electrical signals, triggering the corresponding behavioural responses.^12^

*Helicoverpa assulta* is an oligophagous insect with a relatively limited host‐plant preference, and mainly specializes in members of the Solanaceae, such as tobacco, hot peppers and several *Physalis* species.^23^, ^24^ *H. assulta* is a serious threat to tobacco and peppers in China.^25^ Among the host plants, *H. assulta* females show a strong oviposition preference for tobacco compared with sunflowers, hot peppers, green peppers, aubergines or tomatoes.^7^, ^26^, ^27^ More eggs are found on the leaves than on other parts of the tobacco plant, such as the stems, fruit and flowers.^27^ Chemical cues are important in host selection and female egg‐laying preferences. Previous studies have identified several tobacco volatile components.^28^, ^29^, ^30^, ^31^ Some of these compounds, such as (*E*)‐β‐ocimene, (*Z*)‐3‐hexenyl acetate, nonanal and (*E*)‐β‐caryophyllene, have been reported to be active components because they elicit electroantennogram (EAG) responses in the antennae and/or attract female *H. assulta* moths in a wind tunnel.^31^ All these electrophysiological and behavioural responses have been observed in *H. assulta*, but it is unclear how oviposition signals are received and perceived by the peripheral nervous system of the antennae in female *H. assulta*.

Sixty‐four OR genes have been identified from *H. assulta* antennal transcriptome data,^32^ but few genes have been characterized functionally.^15^, ^33^ In a previous study, functional characterization of an *in vitro* expression system showed that HassOR12 was tuned to general plant volatiles, and its homologous ORs in related heliothine moths showed a conserved function.^15^ The active tobacco volatile nerolidol is recognized by the HassOR40‐neuron line and attracts both sexes of *H. assulta* adults.^33^ However, oviposition preference in female *H. assulta* as an oligophagous herbivore is still not fully understood.

In this study, volatile compounds emitted from tobacco, *Nicotiana tabacum*, were identified using gas chromatography–mass spectrometry (GC–MS) analysis. Electrophysiological responses were detected using EAG recordings, and female *H. assulta* behaviours in response to these volatiles were tested using an oviposition preference assay. Furthermore, the responses of a variety of ORNs in the antennae of mated females were recorded using single sensillum recording (SSR). Importantly, several full‐length OR genes were screened from the antennae of *H. assulta* and further characterized functionally with oviposition‐attractive compounds using a *Xenopus* oocyte expression system. The study uncovered several chemoreception mechanisms that elucidate how volatiles emitted from *N. tabacum* induce oviposition preference in female *H. assulta*.

## 2 MATERIALS AND METHODS

### 2.1 Plants

Seeds of tobacco, *N. tabacum* (var. K326), were obtained from the National Tobacco Cultivation and Physiological and Biochemical Research Base, Henan Province, China. Seedlings were float‐bred in a greenhouse at 20 ± 5 °C and 70 ± 5% relative humidity (RH) under a natural photoperiod. Plants were transplanted to an experimental field (Zhengzhou, Henan Province, China) when the euphylla had expanded to four to five leaves. Tobacco plants at the vegetative growth stage were used to collect the volatiles during the period 2–6 July 2018.

#### 2.1.1 *Odour collection protocol*

Samples of intact tobacco plants at the vegetative growth stage were enclosed in a commercial polyamide roasting bag (Toppits®, Cofresco Frischhalteprodukte, GmbH, Minden, Germany). Charcoal‐filtered air was drawn through the inlet of the bag at a flow rate of 500 mL min^−1^. A glass tube (0.6 cm diameter) containing 100 mg of Tenax TA (mesh size 60–80; Zhengzhou Spectrum Analysis Technology Co., Ltd. Zhengzhou, China) was connected to the outlet of the bag.^34^ After 12 h of collection (20:00 to 08:00 the next morning, at an average temperature of 26 °C), volatiles were eluted from the adsorbent with 2 mL of distilled hexane (chromatography, 98.0%; Merck, Darmstadt, Germany), and the extracts were concentrated to 100 μL using a sample concentrator (MD200‐1; Hangzhou Jingfei Instrument Technology Co. Ltd, Hangzhou, China), then stored at −20 °C before GC–MS analysis. A control sample (empty bag) was also collected in parallel with the odour collection session. Each treatment was conducted in triplicate.

#### 2.1.2 *Coupled GC–MS analysis*

Samples (2 μL) were injected using an autoinjector (G4567A) on a coupled gas chromatograph mass spectrometer (GC–MS ISQ; Thermo Scientific, Waltham, Massachusetts, USA). The injector temperature was 225 °C. The GC was equipped with a DB‐WAX column (30 m × 0.25 mm × 0.25 μm; Agilent Technologies Inc., Santa Clara, CA, USA). The temperature was programmed as follows: 80 °C for 1 min; 5 °C min^−1^ to 150 °C and held for 1 min; 10 °C min^−1^ to 250 °C and held for 5 min. Helium was used as the mobile phase at a constant flow rate of 35 cm s^−1^. Mass spectra were obtained in the electron impact (EI) mode at 70 eV, scanning *m*/*z* 29–400, at 3.8 scans s^−1^.

Major volatile compounds were identified by comparing their mass spectra supplemented with the National Technical Information Services (NTIS) library spectra and confirmed using standard compounds. A compound was considered reasonably identified and assigned a specific chemical name only if the MS library match was > 90%. Synthetic standards were used to further confirm the volatile compounds.

### 2.2 Insects

Larvae of *H. assulta* were originally obtained from a tobacco field at the Xuchang campus of Henan Agricultural University, Henan Province, China, in 2002, and refreshed annually with new wild‐collected individuals. The entire rearing cycle took place in climate chambers at 26 ± 2 °C, 75 ± 5% RH and a 16 : 8 h light/dark photoperiod. Larvae were reared on an artificial diet. Pupae were separated based on their sexual dimorphism. Moths were provided with 10% (w/w) sucrose solution, and 3‐ or 4‐day‐old females were used in the experiments.

#### 2.2.1 *Electroantennogram response*

Mated females (aged 3–4 days) were used for the electrophysiology responses study. The antennae of female moths were cut at the base of the flagellum. The tip was then removed. The basal part of the antenna was inserted into a glass electrode filled with 0.1 Mol L^−1^ KCl solution as a reference electrode. The tip of the antenna was inserted into another electrode as the recoding electrode. A single volatile compound was diluted in hexane at a concentration of 1 μg μL^−1^ (w/v). Ten microlitres of the chemical stimulus dissolved in hexane was dripped onto a filter paper strip (0.5 cm × 5 cm) and inserted in a Pasteur pipette (15 cm long), and 10 μL of hexane was used as a blank control. The filter paper strip loaded in the Pasteur pipette was used only once for each antenna and then removed. The pipette was sealed with Parafilm® between puffs at room temperature. Antennae were stimulated with hexane and each volatile individually in a random order. A purified and humidified air flow of 10 mL s^−1^ blew continuously towards the antenna through a 14‐cm‐long metal tube controller. The outlet of the tube was ~ 1 cm from the antenna. The tip of a Pasteur pipette was inserted into the small hole in the airflow tube, and the odour was carried to the antenna by the airflow. The fixed antennae were exposed to 1 s odour air pulses with an air flow rate of 10 mL s^−1^ and an interval of 30 s between two stimulation pulses, allowing the antenna to recover its normal status. The pulse signals of the antennae were amplified by a 10× AC/DC Headstage preamplifier (Syntech, Hilversum, The Netherlands). Signals were acquired using an intelligent data acquisition controller (IDAC‐4‐USB; Syntech) and sent to a computer and recorded with the Syntech EAG software.^15^ EAG responses were calculated as the differences between the treatment and control; these were standardized as response = (compound response – blank response). Eight biological replicates were tested. Statistical significance was determined at the levels of α = 0.05 and 0.01 using Student–Newman–Keuls tests with IBM SPSS Statistics 19.

#### 2.2.2 *Egg‐laying assay*

After detecting the electrophysiological activity of the volatile compounds, oviposition choice assays were performed to determine which of these compounds induced oviposition preferences in females. Eight newly emerged moths, three females and five males, were kept in a plastic cylindrical container (30 cm in length, 28 cm in diameter). A layer of gauze was placed on top of the plastic container and divided crosswise into four parts. Four agar columns loaded with hexane (control) or single compounds identified from tobacco volatiles were placed on the edges of the four parts of the gauze (Fig. S1). Each odour compound was diluted in hexane, resulting in 0.01 and 0.001 Mol L^−1^ solutions, and the placement was repeated between six and nine times. Each agar column was mixed with 20 μL of hexane or a volatile compound solution.^35^ Containers were rotated 90° clockwise every day. The numbers of eggs on each part of the gauze were counted after 72 h, and the oviposition preference index (OPI) was calculated as OPI = (number of eggs in the treatment − number of eggs in the control)/total number of eggs.^36^, ^37^ OPI data were analysed with two‐factor analysis of variance (ANOVA) and Student–Newman–Keuls tests.

#### 2.2.3 *Single‐sensillum recording*

Oviposition attractants were selected for further study to understand whether these compounds could activate single‐neuron responses. A mated female moth was restrained in a 1 mL plastic pipette with the entire head protruding. The rim of the pipette tip was fixed with dental wax, and one of the antennae was attached to a glass slide with double‐face adhesive tape.^38^

A tungsten wire was inserted into the compound eye of the moth as a reference electrode. The recording electrode was also a tungsten wire that was sharpened electrolytically by repeatedly immersing the tip into a 40% KNO_2_ solution. The recording electrode was attached to an olfactory probe (Syntech) and gently inserted into the base of each trichoid sensillum and basiconic sensillum until a stable electrical signal with a high signal‐to‐noise ratio was obtained. Recordings were performed under a Leica Z16 APO microscope at ×920 magnification. For the chemical stimuli, volatile compounds were diluted in hexane and used to stimulate the antenna using a dose of 1 mg. The solution was dripped onto a filter paper strip (0.8 × 2.6 cm) inserted into a Pasteur pipette (15 cm in length). A flow of purified and humidified air blew continuously towards the antenna through a 15‐cm‐long metal tube controller (Syntech). The fixed antennae were exposed to a 300 ms odour air pulse with an air flow rate of 20 mL s^−1^ delivered through a Pasteur pipette. The preamplifier was set at a gain of 10×. Action potentials were amplified, digitized and displayed on a computer screen using the software package Autospike (Syntech). Response values for specific odour stimuli were calculated as the difference between the spike number observed 1 s before the stimulus delivery point and 1 s after stimulus delivery.^38^, ^39^ Responses were recorded at the basal, middle and top parts of the antennae of ten individuals, and 12 sensilla were recorded for each individual.

### 2.3 RNA extraction and cDNA synthesis

The antennae of 30 *H. assulta* were dissected from 3‐ or 4‐day‐old adults and frozen in liquid nitrogen for preparation. Total RNA extraction was performed with TRIzol reagent following the manufacturer's instructions (Invitrogen, Carlsbad, CA, USA). Total RNA was dissolved in RNase‐free water, and RNA quality was verified by gel electrophoresis. RNA concentration was determined using a Nanodrop ND‐1000 spectrophotometer (NanoDrop Products, Wilmington, DE, USA). cDNA was synthesized with a RevertAid First Strand cDNA Synthesis Kit (Fermentas, Vilnius, Lithuania). The synthesized cDNA was then used as a template to clone the odorant receptor genes of *H. assulta*.

### 2.4 Cloning of OR genes from *H. assulta*

According to the published antennal transcriptome of *H. assulta* and the phylogenetic tree of the ORs,^32^, ^40^ we selected eight full‐length OR sequences, OR9, OR18, OR20, OR23, OR26, OR31, OR52 and OR67, and designed specific primers (Table S1) to clone the full‐length open reading frame (ORF) of OR genes from *H. assulta* using PrimeSTAR HS DNA polymerase (Takara, Dalian, China). The 50 μL polymerase chain reaction (PCR) mixture contained 25 μL PrimeSTAR mix, 1 μL of each primer, 1 μL cDNA template and 22 μL water.

Reactions were carried out under the following conditions: 94 °C for 5 min; 35 cycles of 94 °C for 30 s, 55 °C for 45 s, 72 °C for 1.5 min; and 72 °C for 10 min. The PCR product was separated by electrophoresis on a 1.0% agarose gel, and the sequence was verified after ligation into the cloning vector pEASY‐Blunt (TransGen Biotech, Beijing, China).

### 2.5 Receptor expression in *Xenopus* oocytes and two‐electrode voltage‐clamp electrophysiological recordings

Electrophysiological recording was performed as described previously.^41^, ^42^ The full‐length cDNA sequences of *ORs* were cloned into pT_7_Ts vectors. cRNA was synthesized from linearized vectors with the mMESSAGE mMACHINE T7 kit (Ambion, Austin, TX, USA). Mature healthy oocytes of *Xenopus laevis* were purchased from the Xenopus Resource Center (HangZhou, China). Oocytes were separated using 2 mg mL^−1^ collagenase I for 1 h at room temperature and then washed with washing buffer (96 mm NaCl, 2 mm KCl, 5 mm MgCl_2_ and 5 mm Hepes; pH 7.6 adjusted using NaOH). A mixture of 27.6 ng OR cRNA and 27.6 ng Orco cRNA was microinjected into the oocytes. Injected *Xenopus* oocytes were incubated at 18 °C in incubation medium (1 × Ringer's buffer, 5% dialysed horse serum, 50 μg mL^−1^ tetracycline, 100 μg mL^−1^ streptomycin and 550 μg mL^−1^ sodium pyruvate) for 3–5  days. After incubation, the whole‐cell currents from the injected oocytes were recorded with a two‐electrode voltage clamp. Odorant‐induced currents were recorded with an OC‐725C oocyte clamp (Warner Instruments, Hamden, CT, USA) at a holding potential of −80 mV. The glass electrodes were filled with 3 Mol L^−1^ KCl. The compounds used in recording were presented in random order for 15 s at a flow rate of 8 mL min^−1^. After each stimulus, oocytes were washed in 1× Ringer's buffer at 10 mL min^−1^ until the current returned to the baseline value. Data were acquired and analysed using Digidata 1440A and Pclamp 10.0 software (Axon Instruments Inc., Union City, CA, USA).

## 3 RESULTS

### 3.1 Plant volatile compounds identified from *N. tabacum*

Dozens of volatile chemicals emitted from *N. tabacum* were separated and detected by GC–MS. Volatile compounds were classified as aldehydes, terpenoids, esters, ketones, aromatic compounds or heterocyclic compounds. Some volatile chemicals were confirmed using chemical reference substances in accordance with the results of MS analysis. After removing alkanes and interfering compounds (non‐phytochemicals), 14 chemicals were identified as ‘biologically active’ candidates, including aldehydes (e.g. heptanal and nonanal), terpenoids (e.g. linalool, farnesol, phytol, *trans*‐caryophyllene and caryophyllene oxide), esters (e.g. geranyl isovalerate), aromatic compounds (e.g. phenylacetaldehyde, methylnaphthalene and methyl salicylate), ketones (e.g. solanone and norsolandione) and heterocyclic compounds (e.g. nicotine) (Table 1 and Fig. S2). Some of these chemicals have been reported previously as volatiles of other *N. tabacum* varieties or *Nicotiana* species.^28^, ^29^, ^30^, ^31^, ^43^

**Table 1:** Volatile compounds identified from *Nicotiana tabacum*

Retention time (min)	Compound	Molecular formula	Relative peak area (%)^a^
5.49	Heptanal	C_7_H_14_O	0.22
5.67	Nonanal	C_9_H_18_O	0.11
8.56	Linalool	C_10_H_18_O	0.04
9.69	*trans*‐Caryophyllene	C_15_H_24_	0.19
10.22	Phenylacetaldehyde	C_8_H_8_O	0.63
12.41	Solanone	C_13_H_22_O	4.75
12.54	Farnesol	C_15_H_26_O	0.05
13.01	Methyl salicylate	C_8_H_8_O_3_	0.62
14.53	Methylnaphthalene	C_11_H_10_	0.11
15.07	Nicotine	C_10_H_14_N_2_	0.15
16.82	Phytol	C_20_H_40_O	0.22
17.39	Geranyl isovalerate	C_15_H_26_O_2_	0.03
17.55	Caryophyllene oxide	C_15_H_24_O	0.10
20.02	Norsolandione	C_11_H_19_O_2_	0.15

a Relative peak area indicates the proportion of each peak area in the sum of the peak areas of all compounds.

### 3.2 Electroantennogram response

**Figure 1:**
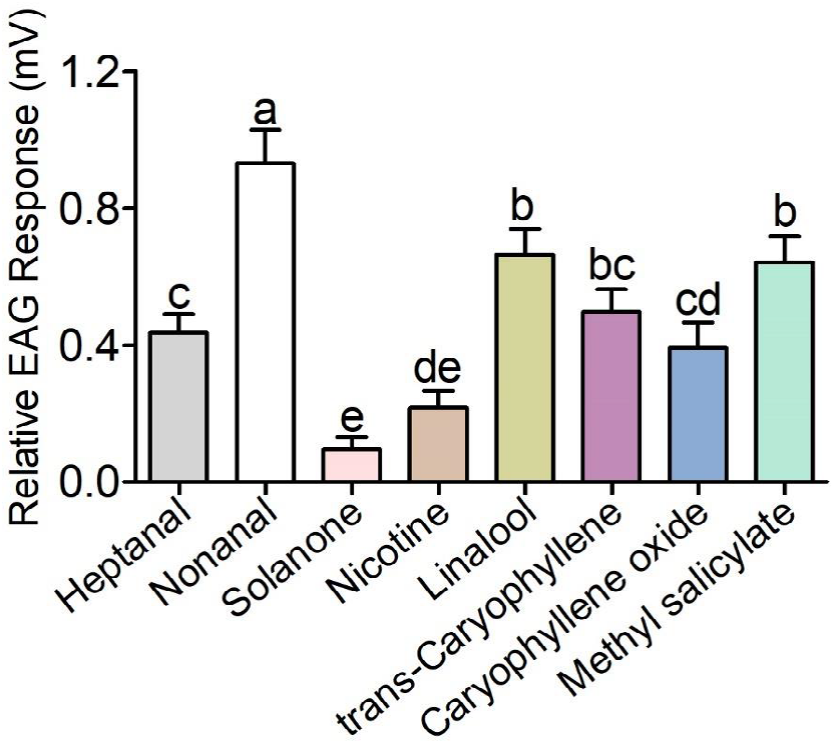
Electroantennogram (EAG) responses of mated *Helicoverpa assulta* female to eight volatile compounds from *Nicotiana tabacum* plants. Different lowercase letters represent the significances of the EAG response to different compounds at the same dose of 10 μg at the 0.01 level. Error bars indicate SEM (*n* = 8).

Eight compounds identified from *N. tabacum* volatiles (Table 1) were chosen to evaluate the EAG responses of female *H. assulta*. The results showed that all the tested compounds induced different EAG responses at the same dose (10 μg) (Fig. 1). The response to nonanal was significantly stronger than the responses to other compounds (*P* < 0.01). Linalool, methyl salicylate, heptanal and *trans*‐caryophyllene induced moderate EAG responses, whereas solanone, nicotine and caryophyllene oxide produced weak responses.

### 3.3 Oviposition preference

**Figure 2:**
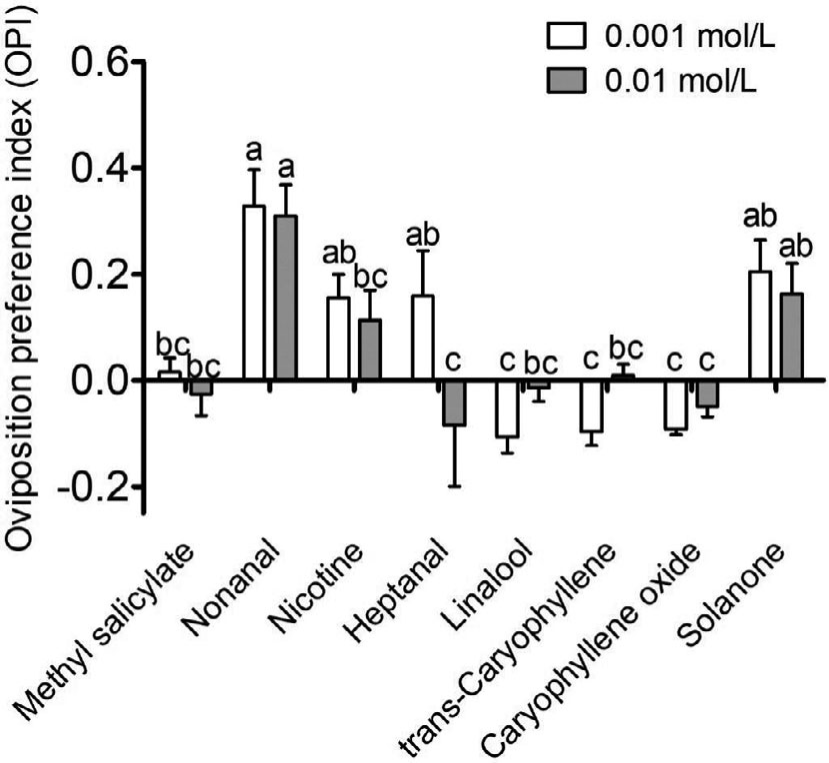
Egg‐laying preferences of female *Helicoverpa assulta* to eight odours at concentrations of 0.001 and 0.01 Mol L^−1^. An oviposition preference index (OPI) was calculated after 72 h of the assay. Error bars indicate SEM (n = 6–9). The OPI differences for the eight compounds were analysed (one‐way ANOVA followed by Student–Newman–Keuls test, *P* < 0.05).

To verify the compounds that elicited egg‐laying behaviours, the OPI of females for the eight compounds tested in the EAG experiment was evaluated at concentrations of 0.001 and 0.01 Mol L^−1^. The results showed that there were significant differences in OPI responses to the tested odorants at each concentration (*P* < 0.05). Nonanal, nicotine, solanone and heptanal at a concentration of 0.001 Mol L^−1^ were significantly preferred for oviposition by *H. assulta*. However, the egg‐laying preference for heptanal disappeared when the concentration increased to 0.01 Mol L^−1^. The other four volatiles, caryophyllene oxide, linalool, *trans*‐caryophyllene and methyl salicylate, did not induce any positive egg‐laying preference at either of the tested concentrations. The OPI for nonanal was significantly higher than for methyl salicylate, linalool, *trans*‐caryophyllene and caryophyllene oxide (*P* < 0.05) (Fig. 2).

### 3.4 Functional characterization of ORNs in response to four bioactive compounds from *N. tabacum*

To test whether the candidate oviposition attractants of female *H. assulta* could activate neurons housed in antennal sensilla, the responses of single neurons in both trichoid and basiconic sensilla were recorded on the antennae of female *H. assulta* using a SSR technique. The preliminary experiments indicated that neurons housed in trichoid sensilla were not activated by nonanal, heptanal, solanone or nicotine (data not shown). Subsequently, numerous ORNs housed in 114 basiconic sensilla of mated female antennae were recorded with the four compounds at a dose of 1 mg. We found that ORNs housed in 75 basiconic sensilla belonging to two morphological classes, so‐called short basiconic (SB) sensilla and long basiconic (LB) sensilla,^33^, ^38^ were activated by the bioactive stimulant nonanal.

**Figure 3:**
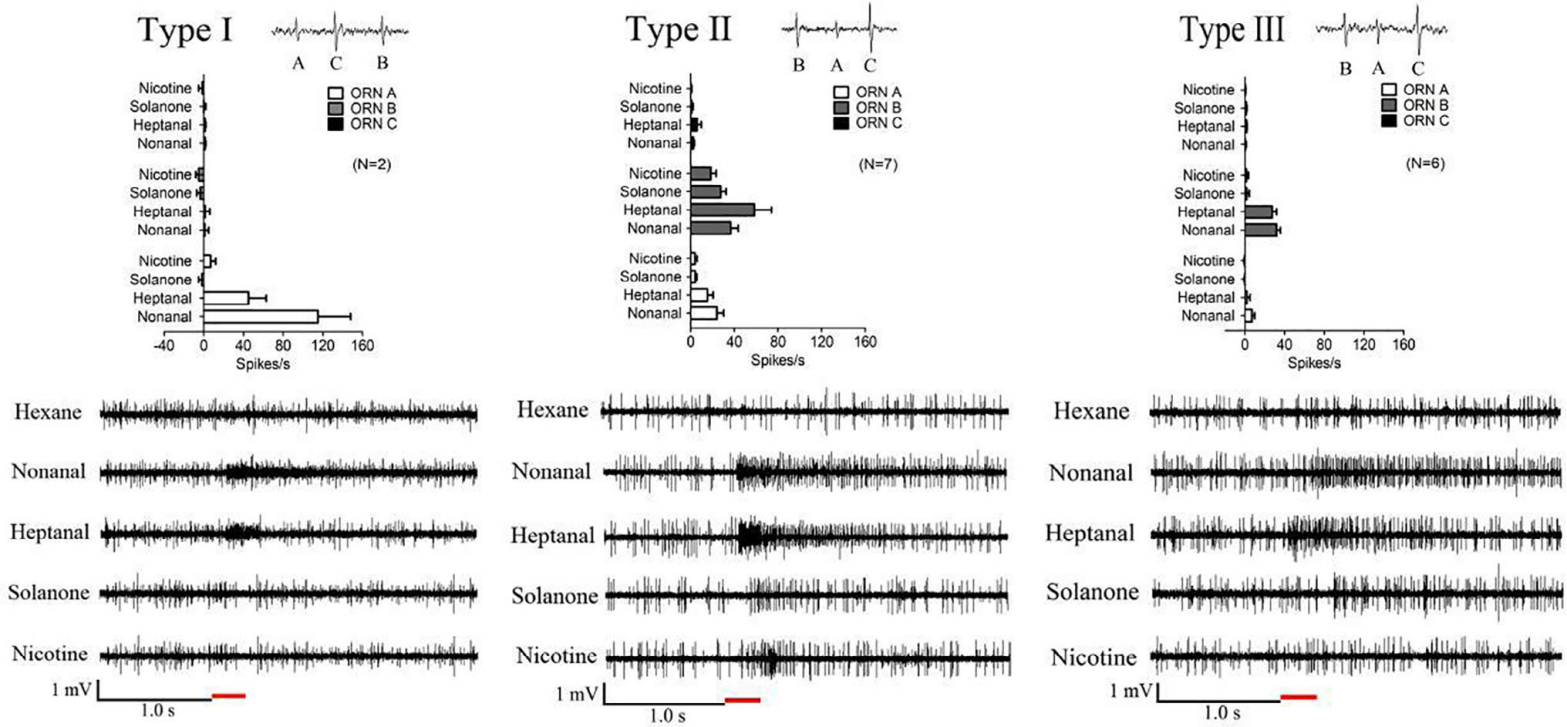
Response profiles of the distinct odorant receptor neurons (ORNs) housed in three types (I, II and III) of short basiconic sensilla (SB) on the antenna of mated *Helicoverpa assulta* females with 1 mg stimulation. The three neurons housed in each sensilla type were named A, B and C. The responses of ORN A in type I were 115 ± 33 spikes s^−1^ for nonanal and 45 ± 18 spikes s^−1^ for heptanal. The ORN A in type II was activated by nonanal and heptanal with responses of 24 ± 6 and 15 ± 5 spikes s^−1^, respectively, and the responses of ORN B were 37 ± 7 for nonanal, 58 ± 16 for heptanal, 27 ± 5 for solanone and 18 ± 5 spikes s^−1^ for nicotine. ORN B in type III was activated by nonanal and heptanal with responses of 32 ± 4 and 27 ± 4 spikes s^−1^, respectively. The red bold line represents 0.3 s odorant stimulation. Error bars indicate SEM (*n* = 2–7).

**Figure 4:**
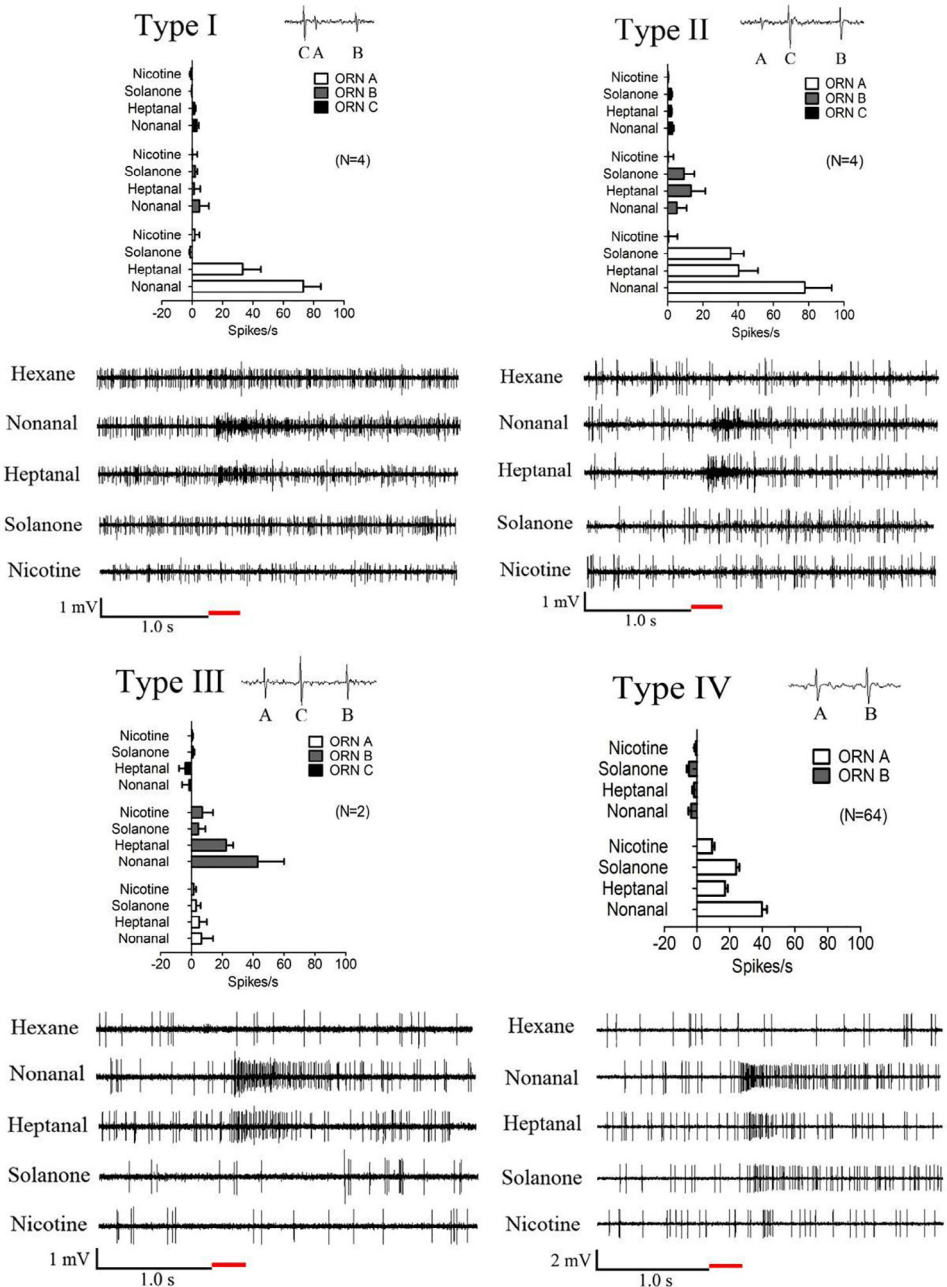
Response profiles of the distinct odorant receptor neurons (ORNs) housed in the four types of long basiconic sensilla (LB) on the antennae of mated *Helicoverpa assulta* females to the test compounds. Type I, II and III each housed three neurons (ORN A, B and C). The responses of ORN A in type I were 74 ± 11 spikes s^−1^ for nonanal and 33 ± 12 spikes s^−1^ for heptanal. For ORN A in type II, 78 ± 15, 40 ± 11 and 36 ± 7 spikes s^−1^ were induced by nonanal, heptanal and solanone, respectively. For ORN A in type III, the responses were 43 ± 17 spikes s^−1^ for nonanal and 23 ± 5 spikes s^−1^ for heptanal. Type IV housed two neurons (ORN A and B). The responses of ORN A to nonanal were 45 ± 3 spikes s^−1^, and those to heptanal, solanone and nicotine were 20 ± 2, 26 ± 2 and 10 ± 2 spikes s^−1^, respectively. The red bold line represents 0.3 s odorant stimulation. Error bars indicate SEM (*n* = 2–64).

The functions of the basiconic sensilla are classified according to sensilla morphology, differentiated responses and neuronal amplitudes. Three types (I, II and III) of SB and LB sensilla were classified, and each type housed three neurons, named A, B and C (Fig. 3 and 4). In addition, a distinct LB sensilla (type IV) containing two neurons, A and B, was identified (Fig. 4). ORN A in type I and ORN B in type III in both SB and LB sensilla were narrowly tuned to nonanal and heptanal, and the neurons in the other types of sensilla tested in this study displayed broad responses to the four bioactive stimulants (Fig. 3 and 4).

### 3.5 Functional characterization of OR67/Orco in the *Xenopus* oocyte expression system

**Figure 5:**
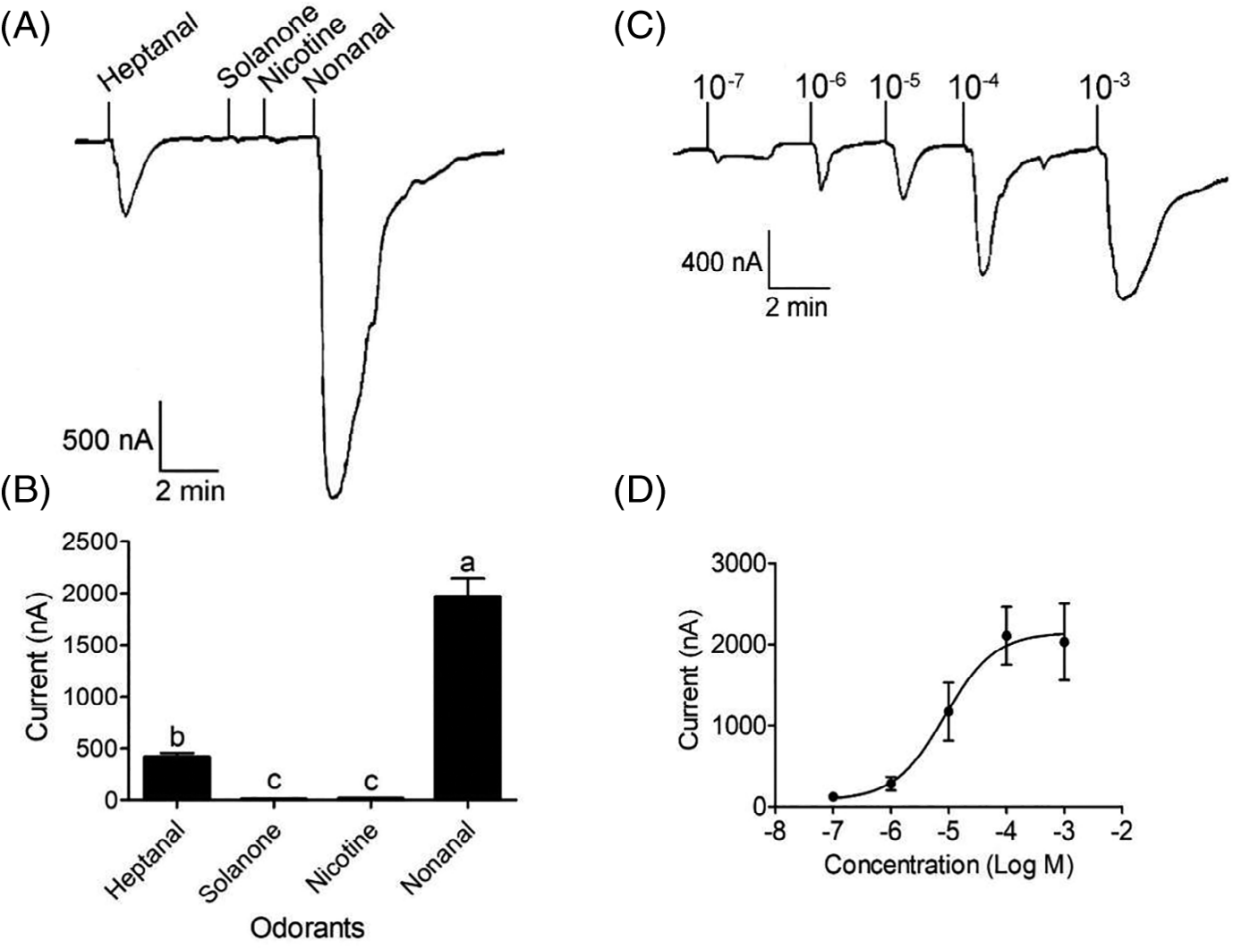
Response profile of HassOR67/Orco to four active tobacco volatiles at a concentration of 1× 10^−4^ Mol L^−1^ (A and B) and the dose–response curve of HassOR67/Orco expressed in *Xenopus* oocytes stimulated by nonanal with a range of concentrations (1 × 10^−7^ to 1 × 10^−3^ m) with an EC_50_ value of 8.486 × 10^−6^ m (C and D). Inward current responses of HassOR67/Orco (A) and tuning curves of HassOR67/Orco (B) to nonanal, heptanal, solanone and nicotine from *Nicotiana tabacum*. The responses activated by the four odorants were significantly different at the 0.01 level (*n* = 7) (B). Error bars indicate SEM (*n* = 8).

Since neurons housed in both SB and LB sensilla could be activated by nonanal, heptanal, solanone and nicotine, it was possible that at least one odorant receptor might evoke responses induced by the four compounds listed above. Hence, the full‐length versions of the eight odorant receptor genes, namely, *HassOR9*, *HassOR18*, *HassOR20*, *HassOR23*, *HassOR26*, *HassOR31*, *HassOR52* and *HassOR67*, were cloned for further functional characterization. All eight HassORs had seven transmembrane domains (Fig. S3). Responses to compounds were recorded using a two‐electrode voltage clamp at a concentration of 1 × 10^−4^ m. The result showed that HassOR67/Orco was mainly tuned to nonanal and its analogue, heptanal. The response of HassOR67/Orco evoked by nonanal at a concentration of 1 × 10^−4^ m was 1963.14 ± 164.44 nA, significantly greater than those induced by heptanal (414.86 ± 37.62 nA), solanone (13.00 ± 1.47 nA) and nicotine (6.57 ± 0.99 nA) (*P* < 0.01) (Fig. 5). Dose–response analysis showed that HassOR67/HassOrco was sensitive to nonanal at concentrations as low as 1 × 10^−7^ m with an 50% effective concentration (EC_50_) of 8.49 × 10^−6^ m (Fig. 5), indicating that nonanal is the main ligand for OR67. No responses to any of the four compounds were obtained from HassOR9, HassOR18, HassOR20, HassOR23, HassOR26, HassOR31 or HassOR52 (Fig. S4).

## 4 DISCUSSION

Moths rely on their remarkably sensitive olfactory organs to recognize odorant molecules from suitable host plants.^1^, ^44^ As an oligophagous herbivore, *H. assulta* also recognizes tobacco volatiles to facilitate host recognition. Hundreds of volatile compounds have been identified from *Nicotiana* species and varieties of *N. tabacum*.^28^, ^29^, ^30^, ^31^ For example, (*E*)‐β‐ocimene, octanal, (*Z*)‐3‐hexenyl acetate, (*Z*)‐3‐hexen‐1‐ol, nonanal, (*Z*)‐3‐hexenyl‐2‐methyl butyrate, decanal, linalool and (*E*)‐β‐caryophyllene, which are volatiles from the flowering stage of *N. tabacum* (var. NC89), elicited consistent EAG responses in female *H. assulta* antennae. A blend of (*E*)‐β‐ocimene, (*Z*)‐3‐hexenylacetate, nonanal and (*E*)‐β‐caryophyllene attracted *H. assulta* females through upwind‐oriented flight.^31^ The chemical (*E*,*E*)‐α‐farnesene, a volatile of tobacco, had an inhibitory effect on the oviposition of female *H. assulta* but an attractive effect on host searching in *Campoletis chlorideae*, the main endoparasitoid of *H. assulta* larvae.^37^ Nerolidol from tobacco volatiles can also attract *H. assulta*.^33^ However, it is not known whether nerolidol is involved in host‐plant selection and oviposition behaviour. In this study, 14 volatile compounds from the vegetative growth stage of *N. tabacum* (var. K326) were identified, some of which could induce EAG responses. Nonanal elicited the strongest EAG response, although it was a relatively less abundant component of the volatiles. By contrast, solanone was an abundant component but induced only a relatively weak response (Table 1). Similar results have been found in other studies. For instance, (*E*)‐β‐caryophyllene was highly abundant in NC89 tobacco volatiles but evoked only a weak EAG response, whereas the strongest EAG response was elicited by nonanal which was present at a much lower concentration.^31^

In general, nonanal is a very common volatile compound in many plants. However, some evidence has shown that nonanal is responsible for essential physiological effects (such as host‐plant selection) in some insects. It has been reported that nonanal is attractive to mated female *Grapholitha molesta* in Y‐tube bioassays.^45^ Nonanal also induced behavioural responses in the source‐contacting and landing of the corn borer *Ostrinia nubilalis* in a wind tunnel.^46^ Most importantly, nonanal is also one of the most abundant volatile components in *Rosa rugose* and *Dianthus caryophyllus*, which are the preferred host plants of female *Frankliniella occidentalis*.^8^ In this study, nonanal, heptanal (at lower concentrations), solanone and nicotine induced obvious oviposition preferences with the largest OPI induced by nonanal. Linalool, *trans*‐caryophyllene, caryophyllene oxide and methyl salicylate showed only slight oviposition effects on mated female *H. assulta*. Hence, our data indicate that nonanal from tobacco plants is one of the most important chemical cues for oviposition site selection by female *H. assulta*.

Neurons housed in trichoid sensilla and basiconic sensilla in females are usually activated by host‐plant volatiles.^33^, ^47^ The functional characterizations of ORNs in heliothine moth species, such as *Heliothis virescens*, *Helicoverpa armigera* and *H. assulta*, have been reported widely for more than a decade. A previous study revealed that some ORN types in different heliothine species responded most strongly to the induced plant volatiles, such as (*E*)‐β‐ocimene, (*E*,*E*)‐α‐farnesene and (3*E*,7*E*)‐4,8,12‐trimethyltrideca‐1,3,7,11‐tetraene, whereas other species responded strongly to geraniol, a common floral volatile.^47^ In this study, the SSR technique was used to detect the existence of ORNs in female antennae that selectively responded to nonanal, heptanal, solanone or nicotine, serving as the active compounds in the egg‐laying preference assay. We found that most of the basiconic sensilla at the basal part of the antennae were more responsive than those in other parts of the antennae (data not shown). Notably, the response profiles of ORNs in basiconic sensilla to nonanal and heptanal were extensive and complex. A high percentage (65.79%) of the neurons in basiconic sensilla were activated by nonanal, including three types of SB and four types of LB, of which the ORN A of type I SB was strongly activated by nonanal with a response of 115.00 ± 33.00 spikes s^−1^ (Fig. 3). These results may explain why nonanal serves as an important chemical cue that influences oviposition preference in female *H. assulta*. In addition, it can be speculated that more than one odorant receptor may be tuned to the responses that were induced by the structurally related plant volatiles nonanal and heptanal. In this study, HassOR67/Orco showed similar response profiles for the functions of ORN A in type I SB (Fig. 3) and LB (Fig. 4), which strongly responded to the structural analogues nonanal and heptanal. Therefore, it is possible that HassOR67/Orco may be expressed in those types of basiconic sensilla, although there is no direct evidence for this relationship to date. Further studies are needed to verify this relationship through *in situ* hybridization assays and CRISPR/Cas9 techniques.

Plant volatiles, such as nerolidol, geranyl acetate, linalool and phenyl ethanol, can act as directional selection or oviposition cues for female moths.^33^, ^48^, ^49^ In this process, ORs are important proteins that are involved in the detection of plant volatiles.^18^, ^49^, ^50^ In this study, we characterized the functions of eight HassORs in the *Xenopus* oocyte expression system and found that the responses of HassOR67 to nonanal and heptanal were sensitive and quite strong. However, the expression level of the *HassOR67* gene was not high in either female or male antennae, as reported in previous antennal transcriptome data.^32^ We also found that the low ratio of ORNs (ORN A in Type I of SB and ORN A in Type I of LB) was specifically tuned to nonanal and heptanal. We could indirectly infer that the *HassOR67*‐expressed neurons may be few in number because the expression level of ORs may be relevant to the quantity of OR‐expressing neurons.

In conclusion, nonanal is a host‐plant volatile of *N. tabacum* involved in the oviposition preference of female *H. assulta*. The recognition mechanism of the peripheral nervous system of the antenna to nonanal was preliminarily elucidated. This will help to develop a new strategy for luring female *H. assulta* and controlling their population.

## Supporting information


**Appendix S1**: Supporting information
