# Supplementary material for: Nonanal modulates oviposition preference in female Helicoverpa assulta (Lepidoptera: Noctuidae) via the activation of peripheral neurons
Source: Pest Manag Sci. 2020 May 10;76(9):3159–67. doi: 10.1002/ps.5870 (PMC7496960; doi:10.1002/ps.5870)
Supplement: Supplementary file 1 — Appendix S1: Supporting information [file PS-76-3159-s001.docx]

**Nonanal modulates oviposition preference in female *Helicoverpa assulta* (Lepidoptera: Noctuidae) via the activation of peripheral neurons**

Chan Wang^ab#^, Guannan Li^a#^, Changjian Miao^a^, Man Zhao^a^, Bing Wang^b^**, Xianru Guo^a^**

a College of Plant Protection, Henan Agricultural University, 450002, Zhengzhou, China

b State Key Laboratory for Biology of Plant Diseases and Insect Pests, Institute of Plant Protection, Chinese Academy of Agricultural Sciences, 100193, Beijing, China.

^#^ These authors contributed equally to this work

** Co-correspondence to: Xianru Guo, E-mail: guoxianru@126.com; Bing Wang, E-mail: bwang@ippcaas.cn.


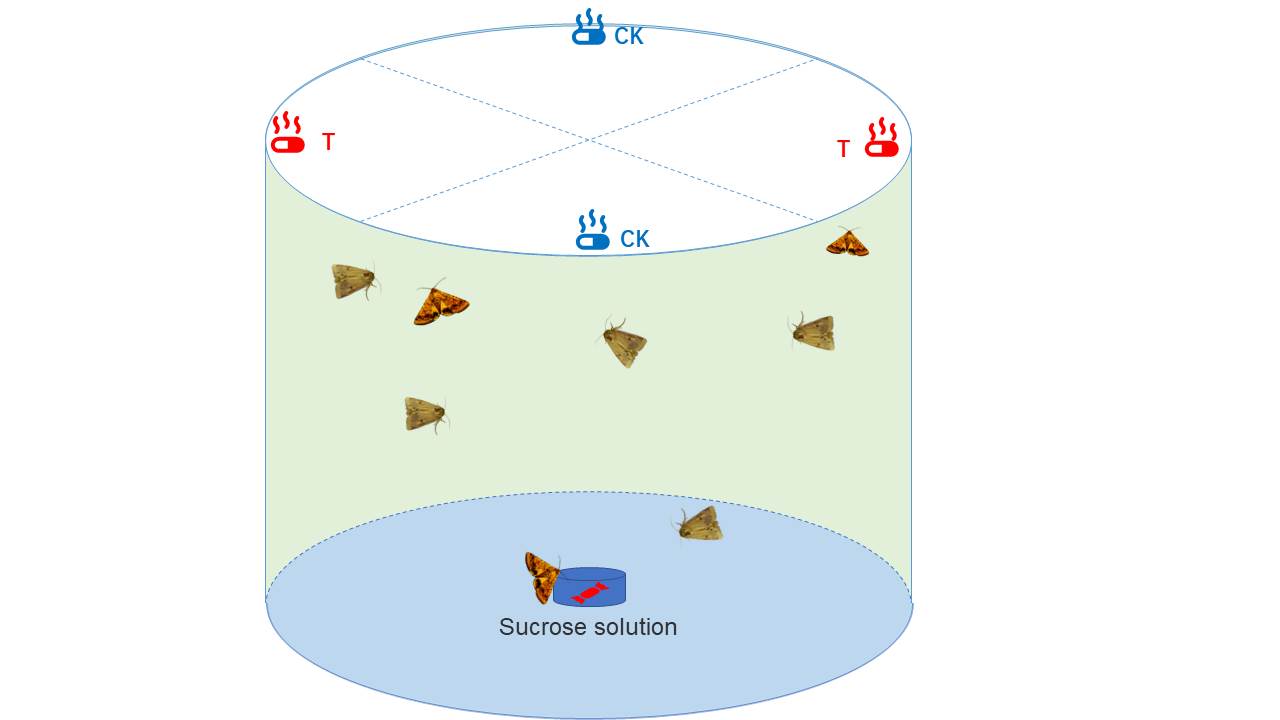


Figure S1. Schematic drawing of the egg-laying assay. CK represent hexane as control; T represent the single compound as treatment; 10% sucrose solution (w:v) was used as diet for the moths


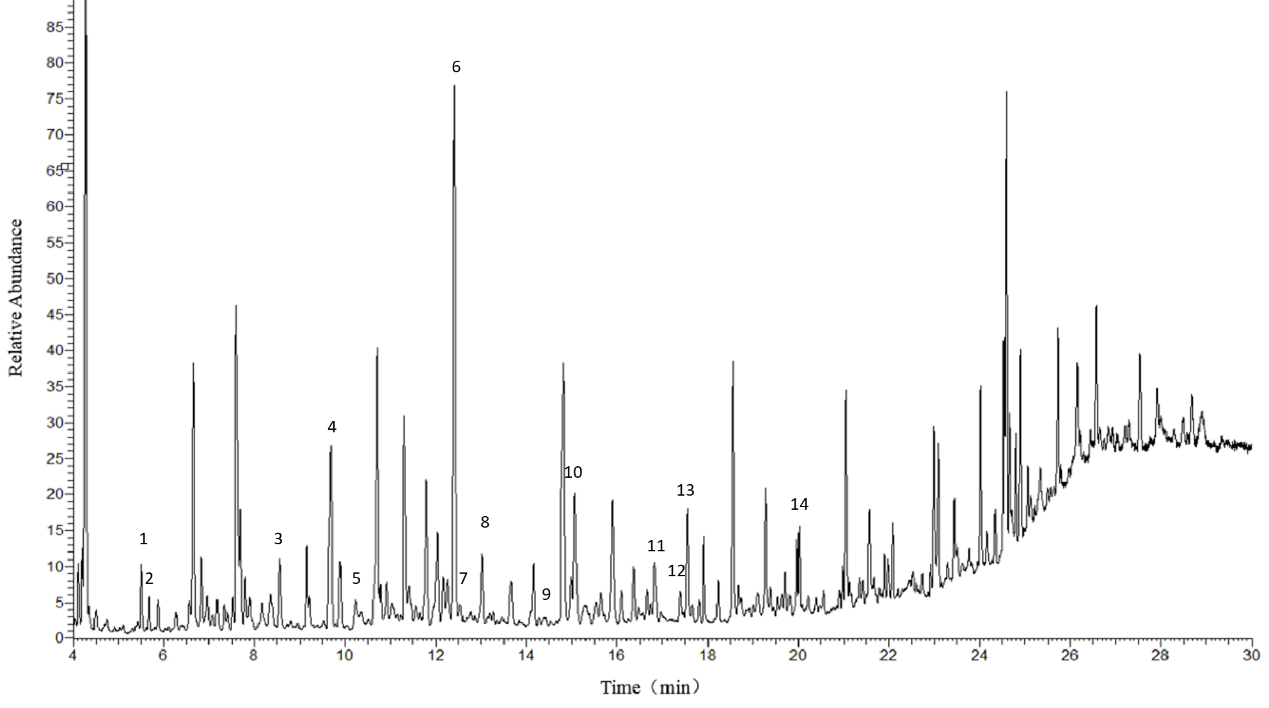


Figure S2. Total ion chromatogram of tobacco volatiles of GC-MS analysis using a DB-WAX column (30 m × 0.25 mm × 0.25 μm). The number labelled in the curve peak indicate the candidate compounds which may be bioactivity to *Helicoverpa assulta* according to the references or its chemical structural formula. Its retention time, peak area, and some information were showed in Table 1


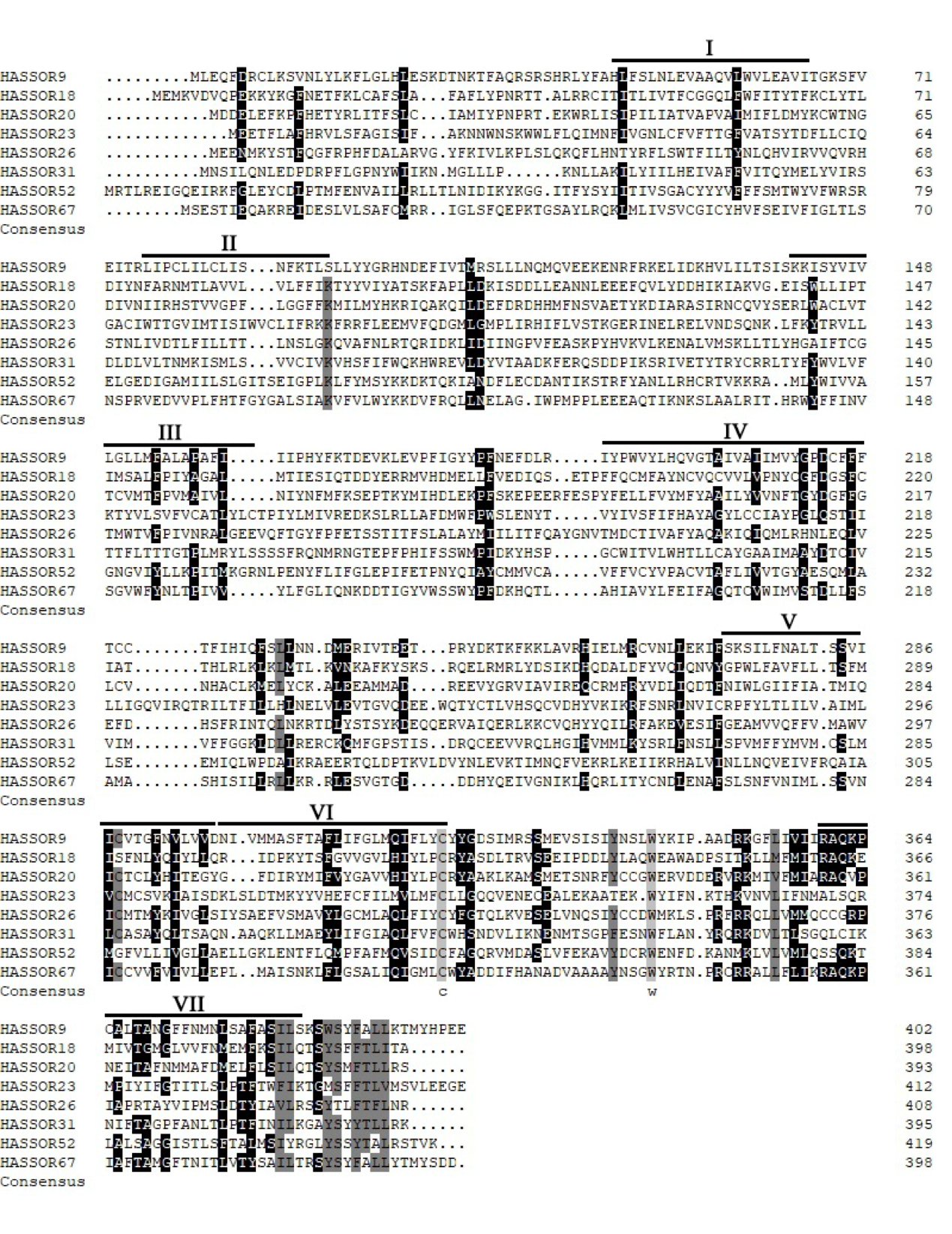


Figure S3. The alignment of amino acid sequence and prediction of seven-span transmembrane domain of HassOR9, HassOR18, HassOR20, HassOR23, HassOR26, HassOR31, HassOR52, and HassOR67


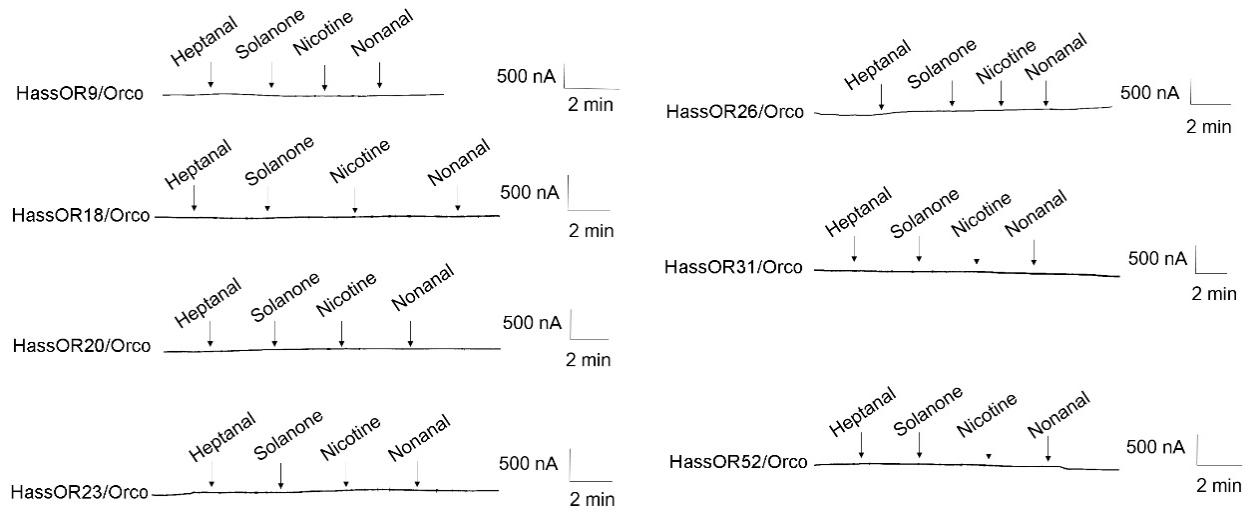


Figure S4. Response profiles of HassOR9, HassOR18, HassOR20, HassOR23, HassOR26, HassOR31, HassOR52/Ocro tuned to the four tobacco volatiles, solanone, nicotine, heptanal and nonanal.

Table S1 Primers for experiments

| **Experiments** | **Primer name** | **Sequence(5’-3’)** |
| --- | --- | --- |
| Cloning of full-length genes | HassOR26-F | ATGGAGGAAAATATGAAATATTCTAC |
|  | HassOR26-R | TTAACGGTTCAAGAACGTGAAC |
|  | HassOR31-F | ATGAATTCAATACTTCAGAACCTAGAA |
|  | HassOR31-R | CTATTTTCTCAGCAGTGTGTAATAACTA |
|  | HassOR52-F | ATGCGGACGTTACGAGAAAT |
|  | HassOR52-R | TACTTGACAGTGGACCGAAGC |
|  | HassOR9-F | ATGCTCGAACAATTTGATAGATGT |
|  | HassOR9-R | TCATTCAGGATGATACATAGTTTTCA |
|  | HassOR18-F | ATGGAAATGAAAGTAGATGTCCAG |
|  | HassOR18-R | TTAAGCAGTAATCAAAGTGAAGAAA |
|  | HassOR20-F | ATGGACGACGAACTAGAATTCAA |
|  | HassOR20-R | TTAAGATCTCAGTAGCGTAAACATTG |
|  | HassOR23-F | ATGGAGGAAACATTTCTTGCATTC |
|  | HassOR23-R | TTAATATTCTCCTTCTTCCAAAACTGA |
|  | HassOR67-F | ATGTCCGAGTCAACAATAGAACAAGC |
|  | HassOR67-R | CTAATCATCACTGTACATTGTATACAGTAATGC |
| Construction of expression vector | HassOR26-F | TCAACTAGT**gccacc**ATGGAGGAAAATATGAAATATTCTAC |
|  | HassOR26-R | TCAGCGGCCGCTTAACGGTTCAAGAACGTGAAC |
|  | HassOR31-F | TCAACTAGT**gccacc**ATGAATTCAATACTTCAGAACCTAGAA |
|  | HassOR31-R | TCAGCGGCCGCCTATTTTCTCAGCAGTGTGTAATAACTA |
|  | HassOR52-F | TCAACTAGT**gccacc**ATGCGGACGTTACGAGAAAT |
|  | HassOR52-R | TCAGCGGCCGCTACTTGACAGTGGACCGAAGC |
|  | HassOR9-F | TCAGGGCCC**gccacc**ATGCTCGAACAATTTGATAGATGT |
|  | HassOR9-R | TCAGCGGCCGCTCATTCAGGATGATACATAGTTTTCA |
|  | HassOR18-F | TCAGGGCCC**gccacc**ATGGAAATGAAAGTAGATGTCCAG |
|  | HassOR18-R | TCAGCGGCCGCTTAAGCAGTAATCAAAGTGAAGAAA |
|  | HassOR20-F | TCAGGGCCC**gccacc**ATGGACGACGAACTAGAATTCAA |
|  | HassOR20-R | TCAGCGGCCGCTTAAGATCTCAGTAGCGTAAACATTG |
|  | HassOR23-F | TCAGGGCCC**gccacc**ATGGAGGAAACATTTCTTGCATTC |
|  | HassOR23-R | TCAGCGGCCGCTTAATATTCTCCTTCTTCCAAAACTGA |
|  | HassOR67-F | TCAGGGCCC**gccacc**ATGTCCGAGTCAACAATAGAACAAGC |
|  | HassOR67-R | TCAGCGGCCGCCTAATCATCACTGTACATTGTATACAGTAATGC |

Note: “F” indicates forward strand; “R” indicates reverse strand; “TCA” indicates protective base, the underlined indicate restriction recognition sites, the bold indicate Kozak sequence.
